# Efficacy and effectiveness of Herpes zoster vaccination in adults with diabetes mellitus: a systematic review and meta-analysis of clinical trials and observational studies

**DOI:** 10.1007/s00592-023-02127-7

**Published:** 2023-06-20

**Authors:** Giovanni Antonio Silverii, Alessandra Clerico, Riccardo Fornengo, Giovanni Gabutti, Valeria Sordi, Ottavia Peruzzi, Silvio Tafuri, Edoardo Mannucci, Ilaria Dicembrini

**Affiliations:** 1 Department of Experimental and Clinical Biomedical Sciences “Mario Serio” Department, University of Florence, Viale Pieraccini, 6, 50139 Florence, Italy; 2 Diabetes Unit, Azienda Sanitaria Città di Torino, Turin, Italy; 3 Diabetes Unit, ASL TO4, Chivasso, Turin, Italy; 4 Coordinator Working Group “Vaccines and Immunization Policies”, Italian Scientific Society of Hygiene, Preventive Medicine and Public Health (SItI), Cogorno, Genoa, Italy; 5 Diabetes Research Institute, IRCCS San Raffaele Hospital, Milan, Italy; 6 Interdisciplinary Department of Medicine, University of Bari Aldo Moro, Bari, Italy

**Keywords:** Diabetes, Herpes zoster-related severe outcome, Efficacy and effectiveness of Herpes zoster vaccination, Herpes zoster vaccine, Meta-analysis

## Abstract

**Aim:**

The risk for Herpes zoster (HZ) and its complications is higher in people with diabetes mellitus (DM). Our aim is to assess efficacy and effectiveness of the currently available live-attenuated zoster vaccine (LZV) and recombinant zoster vaccine (RZV) in adults with DM.

**Methods:**

A Systematic Review and Meta-analysis of clinical trials and observational studies comparing incidence of HZ and its complications in vaccinated and unvaccinated people with DM was performed, on PubMed, Cochrane, Clinical Trials.gov and Embase databases, up to January 15th, 2023. Risk of bias was assessed through the Cochrane Collaboration tool and the Newcastle–Ottawa Scale. The protocol was registered on the PROSPERO website (CRD42022370705).

**Results:**

Only three observational studies reported LZV efficacy and effectiveness in people with DM. A lower risk for HZ infection (MH-OH Ratio 95% CI = 0.52 [0.49, 0.56] was observed, for unadjusted analysis, and 0.51 [0.46, 0.56] for adjusted analysis, both with *P* < 0.00001 and no heterogeneity). No data on LZV safety were reported. A pooled analysis of two trials comparing RZV and placebo, showed a reduced risk for HZ incidence: (95% CI Odds Ratio: 0.09 [0.04–0.19]), with no difference in severe adverse events and mortality.

**Conclusions:**

In our meta-analysis of three observational studies LZV showed a 48% effectiveness in reducing HZ incidence in adults with diabetes whereas in a pooled analysis of two RCTs, RZV showed a 91% efficacy. No data are available on the effects of vaccination on the incidence and severity of HZ-related complications among subjects with diabetes.

**Supplementary Information:**

The online version contains supplementary material available at 10.1007/s00592-023-02127-7.

## Introduction

Herpes zoster (HZ), or shingles, is a neurocutaneous disease determined by the reactivation of a latent varicella zoster virus (VZV) in the dorsal root ganglion, characterized by unilateral radicular pain and a vesicular rash, both usually following a dermatomal pattern [1]. Potential complications of HZ include encephalitis, myelitis, nerve palsies, and, more frequently, postherpetic neuralgia (PHN), defined as pain lasting for more than 3 months after the onset of an HZ infection [2, 3]. PHN may last for years, greatly affecting quality of life, and its management is challenging [4].

The lifetime risk of developing HZ is 25%, but this risk increases sharply after 50 years of age, when two-thirds of HZ cases occur [5, 6]. VZV reactivation has been demonstrated to involve a defect in cell-mediated immunity [7] associated with aging and with diabetes mellitus (DM), leading to increased susceptibility to HZ [8]. Other risk factors include female gender, white race, and recent psychological stress [9].

Two recent observational studies [10, 11] suggested that the association of DM with risk for HZ may disappear after adjusting for age and sex; however, all available meta-analyses of observational studies confirm a significant increase of risk in diabetes mellitus, ranging from 24 to 60%, with an estimated yearly incidence of HZ in people with DM of 7.23–9.36/1.000 [12]–[15]. A further increase in risk of HZ has been observed in older people with diabetes, and in those with diabetes and cardiovascular disease [15]. Patients with diabetes are also at higher risk of complications of HZ, such as acute pain and PNH [16–18], leading to a more frequent use of medication (e.g., opioids) [19], outpatient visits, hospitalizations, sick leave, reduced quality of life and deterioration of glucose control [20].

Two vaccines for HZ are currently available. A live attenuated vaccine (LZV) was first licensed in 2006; it contains the Oka VZV strain (with high antigen content), which has been proved to be safe [21] and effective in a large randomized controlled trial (RCT), reducing the HZ incidence by 51.3%, and PHN by 66.5% [22]; on the other hand, its efficacy is lower in those aged more than 70 years, and it progressively declines over the time. More recently, in 2014, a recombinant subunit zoster vaccine (RZV), containing VZV glycoprotein E and the AS01B adjuvant system was introduced, showing a greater efficacy in two RCTs conducted in the general population: 97.2% reduction of HZ incidence in the older than 50 years, 91.3% in those older than 70 years, without any decrease in efficacy in those older than 80 years [23, 24], nor any decline over 10 years of follow-up [25]; furthermore, a 88.8% reduction in PHN incidence was shown [23, 24]. Based on these results, the Advisory Committee on Immunization Practices USA recommends RZV, rather than LZV, in patients with diabetes older than 50 years [26]. RZV is also being increasingly recommended in national vaccination guidelines across Europe and Canada [27–29].Nevertheless, HZ vaccine coverage is still suboptimal, likely due not only to logistic and economic difficulties [30], but also to the lack of physician recommendations [31], although some virtuous experiences have been reported [32].

A Cochrane review of RCTs performed to date in the general population, has shown that HZ vaccines are efficacious in reducing HZ incidence, and overall safe [33]; however, no systematic review or meta-analysis has explored, to our knowledge, their performance in adults with diabetes, a condition which may theoretically hamper vaccine efficacy [34]. The aim of this Systematic Review and Meta-analysis is therefore to collect the available evidence on efficacy and safety of available HZ vaccines in people with diabetes mellitus. The present work was performed to provide a reliable evidence base for the formulation of a position statement of the Scientific Societies involved.

## Methods

This meta-analysis was performed in according to the criteria of Preferred Reporting Items for Systematic Reviews and Meta Analyses guidelines [35] (Table 1S). Review Protocol was submitted for registration to the PROSPERO website (CRD42022370705).

### Search strategy and selection criteria

A systematic search on PubMed, Cochrane, Clinical Trials.gov and Embase databases was performed, collecting all randomized clinical trials and observational studies performed on humans up to January 15th, 2023. Search string included “Herpes Zoster”. The full search string is reported in Appendix, Table 2S. Further studies were manually searched in references from retrieved papers.

### Inclusion criteria

Full-text publications and conference abstracts showing results of phase II, III and IV RCTs and observational studies were included, provided that:

- Only adults with DM were enrolled, or separate analyses for patients with diabetes were available.
- Efficacy, effectiveness and/or safety of any HZ vaccine, regardless of dose, schedule, preparation, or route of administration, were compared to other HZ vaccines, placebo, or no intervention.
- Reports included at least one of the following outcomes: incidence or severity of HZ or PHN at any time point equal to or longer than 12 months, or for the entire duration of the study; incidence of serious adverse events (SAEs); overall mortality.

Other variables of interest retrieved from selected studies were year of publication, study duration, number, age and sex of participants.

### Data collection

Titles and abstracts were screened independently by eight of the authors, and potentially relevant articles were retrieved in full text. For all published studies, results reported in published papers and supplements were used as the primary source of information; when the required information on protocol or outcomes was not available in the main publication secondary publications were used for retrieval of missing information; whenever needed an attempt at retrieval of missing information was performed consulting the clinicaltrials.gov registry. The identification of relevant abstracts, the selection of studies, and data extraction were performed independently by six of the authors, and conflicts were resolved by a distinct investigator. The risk of bias was assessed independently by two of the authors, and conflicts were resolved through discussion with a third investigator. The Cochrane Collaboration tool [36] was used for RCTs, whereas the Newcastle- Ottawa Scale, available at the https://www.ohri.ca/programs/clinical_epidemiology/oxford.asp website, was adopted for nonrandomized studies; reporting bias was assessed for each main outcome. The GRADE methodology [37] was used to assess the quality of the body of retrieved evidence, using the GRADE pro-GDT software (GRADEpro Guideline Development Tool. McMaster University, 2015).

### Statistical analyses

For each outcome, the number of events and patients enrolled in both arms were retrieved at any time-point for which they were available; when they were not available, or to meta-analyze adjusted analyses, Odds Ratios were retrieved; forest plot were then built collecting all data for each outcome at any given time-point. Between-group Mantel–Haenszel Odds ratio (MH-OR) with 95%, Confidence Intervals (CI) were calculated, on an intention-to-treat basis, for each outcome at any given time-point, using the Wald type confidence interval methods calculator. Heterogeneity was assessed by means of I^2^ statistics, through the Der Simonian and Laird variance estimator. We applied a random-effects model as the primary analysis, because it is more reliable than fixed-effect when the number of component studies is small. If at least six studies were included in a metanalysis for an outcome, a leave-one out analysis was conducted to assess robustness of the synthesized results. If a relevant heterogeneity was detected, subgroup-analyses or meta-regressions were performed taking year of publication, study duration, number, age and sex of participants into account, provided that a sufficient number of studies was available. Funnel plots and Egger regression were examined to estimate possible publication/disclosure bias, if a sufficient number of studies was detected (at least nine). All analyses were performed using Review Manager 5.3.5; The Cochrane Collaboration, 2014, and IBM SPSS Statistics 28.

## Results

The flow research chart is reported in Fig. 1S in the supplementary appendix. The Systematic Search retrieved 12.076 titles, after removing duplicates; of those, 11.969 were excluded after reading titles and abstract. Of the 132 full-text selected, only 5 papers [38]–[42] reported analyses performed on people with diabetes, of which one [42], reported a pooled analysis from two RCTs on RZV, (see below). Therefore, 6 studies were included in this Systematic Review and Meta-analysis.

*Recombinant Zoster Vaccine:* Two randomized clinical trials compared RZV and placebo on people older than 50 [23] and 70 [24] years, respectively. The risk of bias was low (see Table 1 for general Characteristics). A pooled post-hoc analysis of subgroups of patients with diabetes (2,372 patients on active treatment and 2,350 on placebo) enrolled in these two trials has been published [42], showing a significant reduction of HZ (OR [95% CI] was 0.09 [0.04, 0.19]), with incidence of 0.8 and 9.1/1000 patients*years in the RZV and placebo arms, respectively. The quality of Evidence was rated as Moderate with the GRADE Methodology (Table 3S). The incidence of SAEs was similar in the two arms, as it was (15.2 [13.8–16.7]/1.000 patient*years with RZV and 15.4 [14.0–16.9] /1.000 patient*years with placebo. Reported all-cause mortality was 7.3 (6.3–8.4) /1000 patient*years in the RZV arm and 8.3 (7.2–9.4) /1000 patient*years in the placebo arm [42].

**Table 1:** Characteristics of the included randomized controlled studies comparing HZ infection in people with diabetes mellitus with or without prior HZ recombinant subunit zoster vaccine (RZV)

Study	Years of observation	Country	Design	Age Group	Vaccine	Comparator	Patient-years		Risk of Bias						
							Vaccinated	Unvaccinated	A	B	C	D	E	F	G
ZOE-70	2013–2015	UK	RCT	≥ 70 year	RZV	placebo	8723.8	8652.7	L	L	L	L	L	L	L
ZOE-50	2007–2009	USA	RCT	≥ 50 year	RZV	Placebo			L	L	L	L	L	L	L
Hata 2015 UMIN000004771)	2007–2014	JAPAN	RCT	60–70 year	LZV	Placebo	125,038	317,917	L	L	L	L	L	L	L

*A* Random sequence generation (Selection bias), *B* Allocation concealment (selection bias), *C* Blinding of participants and personnel (performance bias), *D* Blinding of outcome assessment (Detection bias), *E* Incomplete outcome data (attrition bias), *F* selective reporting for weight (reporting bias), *G* selective reporting for renal function (reporting bias), *H* other bias, *L* low risk, *U* unknown risk, *H* high risk

**Fig. 1:**
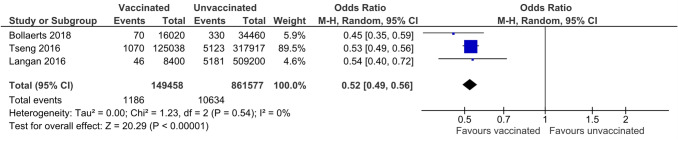
Differences in incidence of Herpes Zoster between vaccinated or unvaccinated (live attenuated vaccine, LZV) patients with diabetes mellitus, unadjusted odds ratio. M-H = Mantel Haenszel; CI = Confidence Intervals

**Fig. 2:**
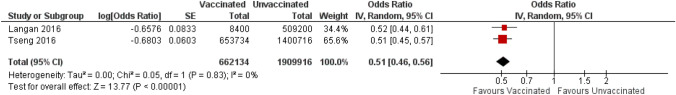
Differences in incidence of Herpes Zoster between vaccinated or unvaccinated (live attenuated vaccine, LZV) patients with diabetes mellitus, adjusted odds ratio. IV = Inverse Variance; SE = Standard Error CI = Confidence Intervals

*Live-attenuated Zoster vaccine:* Only one small RCT performed with the LZV on people with diabetes was retrieved, with only 27 patients per treatment arm, detecting no cases of HZ in the 1-year follow-up [38] (Table 1). Three observational studies, performed on the LZV, provided separate data on people with diabetes mellitus [39]–[41], with a total observation of 149,458 and 861,577 patient*years for vaccinated and unvaccinated individuals, respectively; 1,186 and 10,634 cases of HZ were recorded in vaccinated and unvaccinated individuals. LZV was associated with a significant reduction in risk for HZ in unadjusted analysis (MH-OH Ratio [95% CI] 0.52 [0.49, 0.56], *P* < 0.00001, I^2^ = 0%; Fig. 1). When combining the results on patients with diabetes of the two studies reporting analyses adjusted for some confounding factors [39, 41] (Table 2), MH-OH Ratio [95% CI] was 0.51 [0.46, 0.56], with *P* < 0.00001 and I^2^ = 0% (Fig. 2). The quality of Evidence was rated as Low with the GRADE Methodology (Table 3S).

**Table 2:** Characteristics of the included observational studies comparing HZ infection in people with diabetes mellitus with or without prior HZ live attenuated vaccine (LZV)

Study	Years of observation	Country	Vaccine	Design	Age group	Adjustments	Patient-years		NOS selection				NOS comparability	NOS exposure	
							vaccinated	unvaccinated	1	2	3	4		1	2
Bollaerts 2018	2013–2015	UK	LZV	Retrospective	≥ 70 year	None	34,460	16,020	+	−	+	+	−	+	+
Langan 2016	2007–2009	USA	LZV	Retrospective	≥ 65 year	Age, sex, race, comorbidities, immunosuppression, income	8400	509,200	+	+	+	+	+ +	+	+
Tseng 2016	2007–2014	USA	LZV	Retrospective	≥ 60 year	age, sex, race, healthcare utilization, comorbidities	125,038	317,917	+	+	+	+	+ +	+	+

*NOS* Newcastle–Ottawa Scale

## Discussion

Both LZV and RZV appear to reduce the incidence of HZ in patients with diabetes. However, available data suggest possible differences in efficacy/effectiveness: the incidence of HZ in people with DM is reduced by 95% by RZV, with a number needed to treat (NNT) of 119 for avoiding one case of HZ in one year, whereas the reported reduction with LZV is 48%, with a NNT of 227. Such estimates, however, are derived from studies of different design: results with LZV were obtained meta-analysing three observational studies, whereas those with RZV were reported as a pooled analysis of patient-level data from two randomized controlled trials. The quality of evidence for efficacy of RZV is therefore higher than that for LZV. It is possible that apparent differences in efficacy (95 vs 48%) are at least partly determined by diversities in study design and/or characteristics of enrolled subjects, although the incidence of HZ in control groups of studies on LZV was similar to that of control arm of trials on RZV.

In particular, in observational studies with LZV, those receiving vaccination actively decided to undergo the procedure, whereas in randomized trials vaccination was a play of chance, thus excluding selection bias. It is possible that patients with previous episodes of HZ, or with relatives with a history of recurrent HZ, who could be at greater risk of HZ, were more prone to seek vaccination, thus producing an underestimation of effectiveness of vaccine in observational studies. On the other hand, the more controlled conditions of clinical trials could select subjects who are not fully representative of the general population, generating the possibility of an overestimation of efficacy.

Two network meta-analyses of trials conducted in the general population, showed that the adjuvant RZV is probably superior to LZV, with a greater risk of adverse events at injection sites, but no statistically significant differences for serious adverse events, or death were reported [44, 45]; however, no definitive conclusion can be drawn on this point, since there are no *head to head* comparisons between the two available vaccines in people with DM.

A previous meta-analysis including three observational studies, although limited to elderly subjects only, reported a reduction in the incidence of HZ associated with ZLV [43] similar to that observed in our meta-analysis. Our work is, to our knowledge, the first to systematically assess the efficacy/effectiveness of available HZ vaccines in people with DM [17], with no age limits and including recombinant vaccines.

One of the main goals of vaccination is the prevention of complications of HZ, such as PHN or the rare neurological complications. Diabetes mellitus is associated with an increased risk of both incidence and severity of HZ, including a higher risk for acute and chronic pain [16–18]. Unfortunately, neither studies on LZV or trials with RZV specifically reported the effects of vaccines on HZ complications in people with diabetes. Furthermore, available observational studies on LZV in people with diabetes do not include data mortality and adverse events with LZV, allowing a specific assessment of safety only for RZV. A further limitation is that the presently available data do not allow the assessment of efficacy and safety for subpopulations of diabetic patients stratified for age or comorbidities, preventing the collection of useful information for more targeted recommendations [46].

Overall, available data on people with DM are scarce, which is indeed disappointing given that DM is among the conditions for which a specific recommendation for vaccination has been provided [26, 27] [47]. Such scarcity is a major limitation of our work; on the other hand, the quality of the RCTs and observational studies retrieved is high, and no heterogeneity was detected in our meta-analysis of observational studies. On the other hand, the small number of included studies limits the reliability of I^2^ statistics and prevents the assessment of publication bias.

Recommendations on medical interventions should be based on a careful assessment of risk–benefit and cost-utility ratios. Such assessment requires an estimate of efficacy/effectiveness, such as the one provided by the present meta-analysis. Further data on safety and cost will allow the formulation of properly evidence-based recommendations.

## Supplementary Information

Below is the link to the electronic supplementary material.

Supplementary file1 (DOCX 78 KB)
