# Supplementary material for: Efficacy and effectiveness of Herpes zoster vaccination in adults with diabetes mellitus: a systematic review and meta-analysis of clinical trials and observational studies
Source: Acta Diabetol. 2023 Jun 20;60(10):1343–9. doi: 10.1007/s00592-023-02127-7 (PMC10442285; doi:10.1007/s00592-023-02127-7)
Supplement: Supplementary file 1 — Supplementary file1 (DOCX 78 KB) [file 592_2023_2127_MOESM1_ESM.docx]

**Efficacy and effectiveness of Herpes Zoster Vaccination in adults with Diabetes Mellitus: a Systematic Review and Meta-analysis of clinical trials and observational studies.**

**Supplementary Appendix**

**Table 1S:** PRISMA 2020 Checklist

| **Section and Topic** | **Item #** | | **Checklist item** | | **Location where item is reported** |
| --- | --- | --- | --- | --- | --- |
| **TITLE** | | | | |  |
| Title | 1 | | Identify the report as a systematic review. *Efficacy and effectiveness of Herpes Zoster Vaccination in adults with Diabetes Mellitus: a Systematic Review and Meta-analysis of clinical trials and observational studies.* | | 1 |
| **ABSTRACT** | | | | |  |
| Abstract | 2 | | See the PRISMA 2020 for Abstracts checklist. | | 2 |
| **INTRODUCTION** | | | | |  |
| Rationale | 3 | | Describe the rationale for the review in the context of existing knowledge. *Committee on Immunization Practices USA recommends RZV, rather than LZV, in patients with diabetes older than 50 years . RZV is also being increasingly recommended in national vaccination guidelines across Europe and Canada. Nevertheless, HZ vaccine coverage is still suboptimal, likely due not only to logistic and economic difficulties, but also to the lack of physician recommendations, although some virtuous experiences have been reported.*  *A Cochrane review of RCTs performed to date in the general population, has shown that HZ vaccines are efficacious in reducing HZ incidence, and overall safe; however, no systematic review or meta-analysis has explored, to our knowledge, their performance in adults with diabetes, a condition which may theoretically hamper vaccine efficacy.* | | 3-4 |
| Objectives | 4 | | Provide an explicit statement of the objective(s) or question(s) the review addresses. *The aim of this Systematic Review and Meta-analysis is therefore to collect the available evidence on efficacy and safety of available HZ vaccines in people with diabetes mellitus. The present work was performed to provide a reliable evidence base for the formulation of a position statement of the Scientific Societies involved.* | |  |
| **METHODS** | | | | |  |
| Eligibility criteria | 5 | | Specify the inclusion and exclusion criteria for the review and how studies were grouped for the syntheses. *Inclusion Criteria: Full-text publications and conference abstracts showing results of phase II, III and IV RCTs and observational studies were included, provided that:*  *- only adults with DM were enrolled, or separate analyses for patients with diabetes were available.*  *- efficacy, effectiveness and/or safety of any HZ vaccine, regardless of dose, schedule, preparation, or route of administration, were compared to other HZ vaccines, placebo, or no intervention.*  *- reports included at least one of the following outcomes: incidence or severity of HZ or PHN at any time point equal to or longer than 12 months, or for the entire duration of the study; incidence of serious adverse events (SAEs); overall mortality*. | | 4-5 |
| Information sources | 6 | | Specify all databases, registers, websites, organisations, reference lists and other sources searched or consulted to identify studies. Specify the date when each source was last searched or consulted. *A systematic search on PubMed, Cochrane, Clinical Trials.gov and Embase databases was performed, collecting all randomized clinical trials and observational studies performed on humans up to January 15th, 2023. Search string included “Herpes Zoster”. The full search string is reported in Appendix, Table 2S. Further studies were manually searched in references from retrieved papers.* | | 4 |
| Search strategy | 7 | | Present the full search strategies for all databases, registers and websites, including any filters and limits used. *The full search string is reported in Appendix, Table 2S* | | 4, Table 2S |
| Selection process | 8 | | Specify the methods used to decide whether a study met the inclusion criteria of the review, including how many reviewers screened each record and each report retrieved, whether they worked independently, and if applicable, details of automation tools used in the process. *Titles and abstracts were screened independently by eight of the authors, and potentially relevant articles were retrieved in full text; whenever needed an attempt at retrieval of missing information was performed consulting the clinicaltrials.gov registry. The identification of relevant abstracts, the selection of studies, and data extraction were performed independently by six of the authors, and conflicts were resolved by a distinct investigator* | | 5 |
| Data collection process | 9 | | Specify the methods used to collect data from reports, including how many reviewers collected data from each report, whether they worked independently, any processes for obtaining or confirming data from study investigators, and if applicable, details of automation tools used in the process. . *For all published studies, results reported in published papers and supplements were used as the primary source of information; when the required information on protocol or outcomes was not available in the main publication secondary publications were used for retrieval of missing information;* | | 6 |
| Data items | 10a | | List and define all outcomes for which data were sought. Specify whether all results that were compatible with each outcome domain in each study were sought (e.g. for all measures, time points, analyses), and if not, the methods used to decide which results to collect. *For each outcome, the number of events and patients enrolled in both arms were retrieved at any time-point for which they were available; when they were not available, or to meta-analyze adjusted analyses, Odds Ratios were retrieved;* | | 5 |
|  | 10b | | List and define all other variables for which data were sought (e.g. participant and intervention characteristics, funding sources). Describe any assumptions made about any missing or unclear information. *Other variables of interest retrieved from selected studies were* *year of publication, study duration, number, age and sex of participants.* | | 5 |
| Study risk of bias assessment | 11 | | Specify the methods used to assess risk of bias in the included studies, including details of the tool(s) used, how many reviewers assessed each study and whether they worked independently, and if applicable, details of automation tools used in the process. *risk of bias was assessed by two of the authors independently, and conflicts were resolved through discussion with a third investigator; parameters proposed by the Cochrane Collaboration were used for RCTs, the Newcastle-Ottawa Scale, available at thhttps://www.ohri.ca/programs/clinical_epidemiology/oxford.asp website, was adopted for nonrandomized studies; reporting bias was assessed for each main outcome.* | | 5 |
| Effect measures | 12 | | Specify for each outcome the effect measure(s) (e.g. risk ratio, mean difference) used in the synthesis or presentation of results. *Between-group Mantel-Haenszel Odds ratio (MH-OR) with 95%, Confidence Intervals (CI) were calculated, on an intention-to-treat basis, for each outcome at any given time-point, using the Wald type confidence interval methods calculatorWe applied a random-effects model as the primary analysis, because it is more reliable than fixed-effect when the number of component studies is small. Funnel plots and Egger regression were examined to estimate possible publication/disclosure bias, if a sufficient number of studies was detected (at least nine).* | | 5 |
| Synthesis methods | 13a | | Describe the processes used to decide which studies were eligible for each synthesis (e.g. tabulating the study intervention characteristics and comparing against the planned groups for each synthesis (item #5)). *For all published studies, results reported in published papers and supplements were used as the primary source of information; when the required information on protocol or outcomes was not available in the main publication secondary publications were used for retrieval of missing information; whenever needed an attempt at retrieval of missing information was performed consulting the clinicaltrials.gov registry. The identification of relevant abstracts, the selection of studies, and data extraction were performed independently by six of the authors, and conflicts were resolved by a distinct investigator.* | | 5 |
|  | 13b | | Describe any methods required to prepare the data for presentation or synthesis, such as handling of missing summary statistics, or data conversions. *For each outcome, the number of events and patients enrolled in both arms were retrieved at any time-point for which they were available; when they were not available, or to meta-analyze adjusted analyses, Odds Ratios were retrieved; forest plot were then built collecting all data for each outcome at any given time-point.* | | 5 |
|  | 13c | | Describe any methods used to tabulate or visually display results of individual studies and syntheses. *; forest plot were then built collecting all data for each outcome at any given time-point.* | | 5 |
|  | 13d | | Describe any methods used to synthesize results and provide a rationale for the choice(s). If meta-analysis was performed, describe the model(s), method(s) to identify the presence and extent of statistical heterogeneity, and software package(s) used. *All analyses were performed using Review Manager 5.3.5; The Cochrane Collaboration, 2014, and IBM SPSS Statistics 28.* | | 6 |
|  | 13e | | Describe any methods used to explore possible causes of heterogeneity among study results (e.g. subgroup analysis, meta-regression). *Heterogeneity was assessed by means of I2 statistics, through the Der Simonian and Laird variance estimator. If a relevant heterogeneity was detected, subgroup-analyses or meta-regressions were performed taking year of publication, study duration, number, age and sex of participants into account, provided that a sufficient number of studies was available.* | | 5 |
|  | 13f | | Describe any sensitivity analyses conducted to assess robustness of the synthesized results. *If at least six studies were included in a metanalysis for an outcome, a leave-one out analysis was conducted to assess robustness of the synthesized results.* | |  |
| Reporting bias assessment | 14 | | *Describe any methods used to assess risk of bias due to missing results in a synthesis (arising from reporting biases). results reported in published papers and supplements were used as the primary source of information; when the required information on protocol or outcomes was not available in the main publication secondary publications were used for retrieval of missing information; whenever needed an attempt at retrieval of missing information was performed consulting the clinicaltrials.gov registry. reporting bias was assessed for each main outcome.* | | 5 |
| Certainty assessment | 15 | | Describe any methods used to assess certainty (or confidence) in the body of evidence for an outcome. *The GRADE methodology was used to assess the quality of the body of retrieved evidence, using the GRADE pro-GDT software (GRADEpro Guideline Development Tool. McMaster University, 2015)* | | 5 |
| **RESULTS** | | | | |  |
| Study selection | 16a | | Describe the results of the search and selection process, from the number of records identified in the search to the number of studies included in the review, ideally using a flow diagram. *The flow research chart is reported in figure 1S in the supplementary appendix. The Systematic Search retrieved 12.076 titles, after removing duplicates; of those, 11.969 were excluded after reading titles and abstract. Of the 132 full-text selected, only 5 papers reported analyses performed on people with diabetes, of which one, reported a pooled analysis from two RCTs on RZV, (see below). Therefore, 6 studies were included in this Systematic Review and Meta-analysis.* | | 6 Figure 1S |
|  | 16b | | Cite studies that might appear to meet the inclusion criteria, but which were excluded, and explain why they were excluded. Only one small RCT performed with the LZV on people with diabetes was retrieved, with only 27 patients per treatment arm, detecting no cases of HZ in the 1-year follow-up | | 6, Table 1,2 |
| Study characteristics | 17 | | Cite each included study and present its characteristics. Recombinant Zoster Vaccine: Two randomized clinical trials compared RZV and placebo on people older than 50 [23] and 70 [24] years, respectivelyA pooled post-hoc analysis of subgroups of patients with diabetes (2,372 patients on active treatment and 2,350 on placebo) enrolled in these two trials has been published [42], showing a significant reduction of HZ (OR [95% CI] was 0.09 [0.04, 0.19]), with incidence of 0.8 and 9.1/1000 patients*years in the RZV and placebo arms, respectively. The quality of Evidence was rated as Moderate with the GRADE Methodology (Table 2S). The incidence of SAEs was similar in the two arms, as it was (15.2 [13.8–16.7]/1.000 patient*years with RZV and 15.4 [14.0–16.9] /1.000 patient*years with placebo. Reported all-cause mortality was 7.3 (6.3–8.4) /1000 patient*years in the RZV arm and 8.3 (7.2–9.4) /1000 patient*years in the placebo arm [42].  Live-attenuated Zoster vaccine: Only one small RCT performed with the LZV on people with diabetes was retrieved, with only 27 patients per treatment arm, detecting no cases of HZ in the 1-year follow-up [38] (Table 1). Three observational studies, performed on the LZV, provided separate data on people with diabetes mellitus [39]–[41], with a total observation of 149,458 and 861,577 patient*years for vaccinated and unvaccinated individuals, respectively; 1,186 and 10,634 cases of HZ were recorded in vaccinated and unvaccinated individuals. LZV was associated with a significant reduction in risk for HZ in unadjusted analysis (MH-OH Ratio [95% CI] 0.52 [0.49, 0.56], P < 0.00001, I2=0%; Figure 1). When combining the results on patients with diabetes of the two studies reporting analyses adjusted for some confounding factors [39], [41] (Table 1), MH-OH Ratio [95% CI] was 0.51 [0.46, 0.56], with P < 0.00001 and I2=0% (Figure 2). The quality of Evidence was rated as Low with the GRADE Methodology (Table 2S). | | 6, Table 1,2 |
| Risk of bias in studies | 18 | | Present assessments of risk of bias for each included study. . The risk of bias was low (see Table 1 for general Characteristics). | | 6, Table 1,2 |
| Results of individual studies | 19 | | For all outcomes, present, for each study: (a) summary statistics for each group (where appropriate) and (b) an effect estimate and its precision (e.g. confidence/credible interval), ideally using structured tables or plots. | | 6,7 Figure 1,2 |
| Results of syntheses | 20a | | For each synthesis, briefly summarise the characteristics and risk of bias among contributing studies. | |  |
|  | 20b | | Present results of all statistical syntheses conducted. If meta-analysis was done, present for each the summary estimate and its precision (e.g. confidence/credible interval) and measures of statistical heterogeneity. If comparing groups, describe the direction of the effect. | | 6,7 Figure 1,2 |
|  | 20c | | Present results of all investigations of possible causes of heterogeneity among study results. | | 6,7 Figure 1,2 |
|  | 20d | | Present results of all sensitivity analyses conducted to assess the robustness of the synthesized results. | |  |
| Reporting biases | 21 | | Present assessments of risk of bias due to missing results (arising from reporting biases) for each synthesis assessed. | | 6, Table 1,2 |
| Certainty of evidence | 22 | | Present assessments of certainty (or confidence) in the body of evidence for each outcome assessed. | | 6,7 Figure 1,2 |
| **DISCUSSION** | | | | |  |
| Discussion | 23a | | Provide a general interpretation of the results in the context of other evidence. *Both LZV and RZV appear to reduce the incidence of HZ in patients with diabetes. However, available data suggest possible differences in efficacy/effectiveness: the incidence of HZ in people with DM is reduced by 95% by RZV, with a number needed to treat (NNT) of 119 for avoiding one case of HZ in one year, whereas the reported reduction with LZV is 48%, with a NNT of 227. Such estimates, however, are derived from studies of different design: results with LZV were obtained meta-analysing three observational studies, whereas those with RZV were reported as a pooled analysis of patient-level data from two randomized controlled trials. The quality of evidence for efficacy of RZV is therefore higher than that for LZV. It is possible that apparent differences in efficacy (95 vs 48%) are at least partly determined by diversities in study design and/or characteristics of enrolled subjects, although the incidence of HZ in control groups of studies on LZV was similar to that of control arm of trials on RZV. Two network meta-analyses of trials conducted in the general population, showed that the adjuvant RZV is probably superior to LZV, with a greater risk of adverse events at injection sites, but no statistically significant differences for serious adverse events, or death were reported [44], [45]; however, no definitive conclusion can be drawn on this point, since there are no head to head comparisons between the two available vaccines in people with DM.* | | 7 |
|  | 23b | | Discuss any limitations of the evidence included in the review. *Overall, available data on people with DM are scarce, which is indeed disappointing given that DM is among the conditions for which a specific recommendation for vaccination has been provided [26], [27] [47]. Such scarcity is a major limitation of our work; on the other hand, the quality of the RCTs and observational studies retrieved is high, and no heterogeneity was detected in our meta-analysis of observational studies.* | | 8 |
|  | 23c | | Discuss any limitations of the review processes used. *On the other hand, the small number of included studies limits the reliability of I2 statistics and prevents the assessment of publication bias.* | | 8 |
|  | 23d | | Discuss implications of the results for practice, policy, and future research. *Recommendations on medical interventions should be based on a careful assessment of risk-benefit and cost-utility ratios. Such assessment requires an estimate of efficacy/effectiveness, such as the one provided by the present meta-analysis. Further data on safety and cost will allow the formulation of properly evidence-based recommendations.* | | 8 |
| **OTHER INFORMATION** | | | | |  |
| Registration and protocol | 24a | | Provide registration information for the review, including register name and registration number, or state that the review was not registered. *Review Protocol was submitted for registration to the PROSPERO website (CRD42022370705).* | | 4 |
|  | 24b | | Indicate where the review protocol can be accessed, or state that a protocol was not prepared. *Review Protocol was submitted for registration to the PROSPERO website (CRD42022370705).* | | 4 |
|  | 24c | | Describe and explain any amendments to information provided at registration or in the protocol. NA | |  |
| Support | 25 | | Describe sources of financial or non-financial support for the review, and the role of the funders or sponsors in the review. *Funding and data transparency This research was performed as a part of the institutional activity of the units, with no specific funding.* | | 8 |
| Competing interests | 26 | | Declare any competing interests of review authors. *Potential Conflicts of interest: GG declares grants from Sanofi Pasteur MSD, Pfizer, GSK Biologicals SA, Sanofi Pasteur, MSD Italy, Emergent BioSolutions, Moderna and Seqirus for taking part to advisory boards, expert meetings, for acting as speaker and/or organizer of meetings/congresses and as principal investigator and chief of O.U. in RCTs. All the others authors have no conflict of interest to disclose directly related to this manuscript.* | | 8 |
| Availability of data, code and other materials | 27 | | Report which of the following are publicly available and where they can be found template data collection forms; *data extracted from included studies; data used for all analyses; analytic code; any other materials used in the review. The corresponding author had full access to all the data in the study and had final responsibility for the decision to submit for publication.* | | 9 |
| **Section and Topic** | | **Item #** | | **Checklist item PRISMA 2020 for Abstracts Checklist** | **Reported (Yes/No)** |
| **TITLE** | | | | |  |
| Title | | 1 | | Identify the report as a systematic review. Efficacy and effectiveness of Herpes Zoster Vaccination in adults with Diabetes Mellitus: a Systematic Review and Meta-analysis of clinical trials and observational studies. | yes |
| **BACKGROUND** | | | | |  |
| Objectives | | 2 | | Provide an explicit statement of the main objective(s) or question(s) the review addresses. Our aim is to assess efficacy and effectiveness of the currently available live-attenuated zoster vaccine (LZV) and recombinant zoster vaccine (RZV) in adults with DM | yes |
| **METHODS** | | | | |  |
| Eligibility criteria | | 3 | | Specify the inclusion and exclusion criteria for the review. A Systematic Review and Meta-analysis of clinical trials and observational studies comparing incidence of HZ and its complications in vaccinated and unvaccinated people with DM was performed | yes |
| Information sources | | 4 | | Specify the information sources (e.g. databases, registers) used to identify studies and the date when each was last searched. : A Systematic Review and Meta-analysis of clinical trials and observational studies comparing incidence of HZ and its complications in vaccinated and unvaccinated people with DM was performed, on PubMed, Cochrane, Clinical Trials.gov and Embase databases, up to January 15th, 2023. | yes |
| Risk of bias | | 5 | | Specify the methods used to assess risk of bias in the included studies. Risk of bias was assessed through the Cochrane Collaboration tool and the Newcastle-Ottawa Scale. | yes |
| Synthesis of results | | 6 | | Specify the methods used to present and synthesise results. (in results section) | yes |
| **RESULTS** | | | | |  |
| Included studies | | 7 | | Give the total number of included studies and participants and summarise relevant characteristics of studies. Only three observational studies reported LZV efficacy and effectiveness in people with DM | yes |
| Synthesis of results | | 8 | | Present results for main outcomes, preferably indicating the number of included studies and participants for each. If meta-analysis was done, report the summary estimate and confidence/credible interval. If comparing groups, indicate the direction of the effect (i.e. which group is favoured). Only three observational studies reported LZV efficacy and effectiveness in people with DM. A lower risk for HZ infection (MH-OH Ratio 95% CI= 0.52 [0.49, 0.56] was observed in observational studies, for unadjusted analysis, and 0.51 [0.46, 0.56] for adjusted analysis, both with p< 0.00001 and no heterogeneity; no data on LZV safety were reported. A pooled analysis of two trials comparing RZV and placebo, showed a reduced risk for HZ incidence: (95% CI Odds Ratio: 0.09 [0.04-0.19]), with no difference in severe adverse events and mortality. | yes |

**Table 2S:** Information on search string,

| **PUBMED** Search: (("herpes zoster"[MeSH Terms] OR ("herpes"[All Fields] AND "zoster"[All Fields]) OR "herpes zoster"[All Fields] OR ("herpesviridae"[MeSH Terms] OR "herpesviridae"[All Fields] OR "herpesvirus"[All Fields]) OR ("herpes zoster"[MeSH Terms] OR ("herpes"[All Fields] AND "zoster"[All Fields]) OR "herpes zoster"[All Fields] OR "shingles"[All Fields] OR "shingle"[All Fields]) OR ("herpes zoster"[MeSH Terms] OR ("herpes"[All Fields] AND "zoster"[All Fields]) OR "herpes zoster"[All Fields] OR "zoster"[All Fields]) OR ("herpesvirus 3, human"[MeSH Terms] OR "human herpesvirus 3"[All Fields] OR "varicella"[All Fields] OR "chickenpox"[MeSH Terms] OR "chickenpox"[All Fields] OR "varicellae"[All Fields]) OR "varicellovir*"[All Fields] OR ("herpesvirus 3, human"[MeSH Terms] OR "human herpesvirus 3"[All Fields] OR "hhv3"[All Fields]) OR ("herpesvirus 3, human"[MeSH Terms] OR "human herpesvirus 3"[All Fields] OR "hhv 3"[All Fields]) OR ("post herp*"[All Fields] AND ("neuralgia"[MeSH Terms] OR "neuralgia"[All Fields] OR "neuralgias"[All Fields])) OR "phn"[All Fields])) AND ("vaccin*"[All Fields] OR "immuni*"[All Fields] OR "inocul*"[All Fields] OR ("herpes zoster vaccine"[MeSH Terms] OR ("herpes"[All Fields] AND "zoster"[All Fields] AND "vaccine"[All Fields]) OR "herpes zoster vaccine"[All Fields] OR "zostavax"[All Fields]) OR ("glycoprotein e varicella zoster virus"[Supplementary Concept] OR "glycoprotein e varicella zoster virus"[All Fields] OR "varicella zoster virus glycoprotein e"[All Fields] OR "shingrix"[All Fields]))) AND (humans[Filter]) |
| --- |
| **Embase:** (' herpes zoster '/exp OR herpes zoster OR ' shingles '/exp OR shingles OR ' human herpesvirus 3 '/exp OR human herpesvirus 3 OR varicella zoster) AND ('diabetes mellitus'/exp OR 'diabetes mellitus') AND ('mortality'/exp OR mortality OR 'morbidity'/exp OR morbidity OR 'efficacy'/exp OR 'efficacy' OR 'disease predisposition'/exp OR 'disease predisposition' OR vaccine OR 'vaccine efficacy' OR |
| **Clinicaltrials.gov:** vaccine AND (herpes zoster OR shingles) AND Diabetes |
| **Cochrane database** (‘herpes zoster' OR ‘shingles’) AND ('diabetes’) |


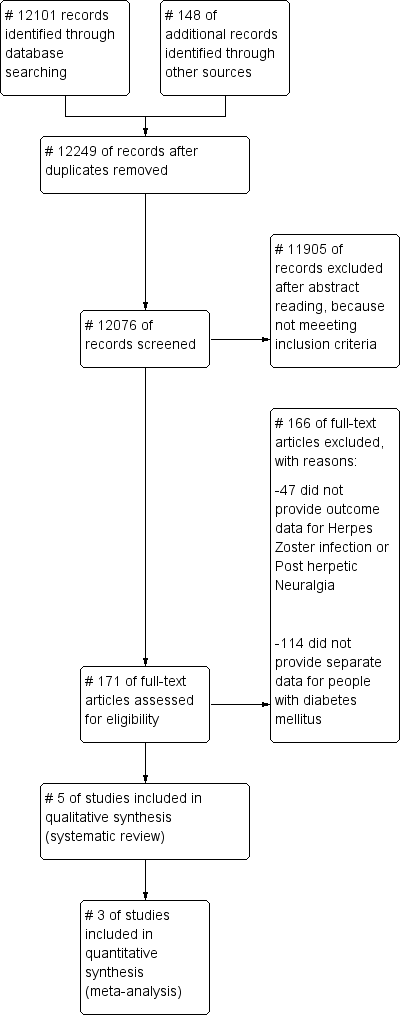


**Figure 1S:** Flow research chart

**Table 3S:** GRADE quality of evidence assessment table

| **Certainty assessment** | | | | | | | **№ of patients** | | **Effect** | | **Certainty** | **Importance** |
| --- | --- | --- | --- | --- | --- | --- | --- | --- | --- | --- | --- | --- |
| **№ of studies** | **Study design** | **Risk of bias** | **Inconsistency** | **Indirectness** | **Imprecision** | **Other considerations** | **Herpes Zoster Recombinant Vaccine** | **Placebo** | **Relative (95% CI)** | **Absolute (95% CI)** |  |  |
| **New outcome** | | | | | | | | | | | | |
| 2 | randomised trials | not serious | not serious | serious^a^ | serious^b^ | strong association | 7/8723.8 (0.1%) | 80/8652.7 (0.9%) | **OR 0.09** (0.04 to 0.19) | **8 fewer per 1.000** (from 9 fewer to 7 fewer) | ⨁⨁⨁◯ Moderate |  |
|  |  |  |  |  |  |  |  |  |  |  |  |  |
| 3 | observational studies | not serious | not serious | serious^a^ | serious^b^ | none | 1186/149458 (0.8%) | 10634/861577 (1.2%) | **OR 0.52** (0.49 to 0.56) | **6 fewer per 1.000** (from 6 fewer to 5 fewer) | ⨁⨁◯◯ Low |  |

**CI:** confidence interval; **OR:** odds ratio

#### Explanations

a. only data on HZ incidence are available

b. small number of patients enrolled, small number of studies
